# Exploration of the association between FOXE1 gene polymorphism and differentiated thyroid cancer: a meta-analysis

**DOI:** 10.1186/s12881-018-0604-y

**Published:** 2018-05-22

**Authors:** Yong-Hui Chen, Ying-Qiang Zhang

**Affiliations:** 1 Department of Nuclear Medicine, Peking Union Medical College (PUMC) Hospital, Chinese Academy of Medical Sciences and Peking Union Medical College, Beijing, 100730 China; 2 Beijing Key Laboratory of Molecular Targeted Diagnosis and Therapy in Nuclear Medicine, Beijing, 100730 China

**Keywords:** Gene polymorphism, FOXE1, Differentiated thyroid cancer

## Abstract

**Background:**

Several association analyses and linkage researches indicated that inherited genetic variations effectively influence differentiated thyroid carcinogenesis.

**Methods:**

The results from 15 published studies on differentiated thyroid carcinoma (DTC) were combined. The genetic model included rs965513, rs944289 and rs1867277. Meta-analyses were performed and cochran’s χ^2^ based Q-statistic and I^2^ test were performed to assess heterogeneity using STATA software.

**Results:**

Significant results were noticed for rs965513(Odds Ratio(OR) = 1.162(1.117, 1.208)), rs944289(OR = 1.082(1.035, 1.131)) and rs1867277(OR = 1.415(1.324, 1.512)). In the subgroup analysis by ethnicity, rs965513 polymorphism conferred that risk of Caucasians (OR = 1.168(1.122, 1.215)) was more than that of East Asians of 1.35 (OR = 0.897(0.680, 1.193)).

**Conclusion:**

This meta-analysis revealed that common variations of FOXE1 (rs965513, rs944289 and rs1867277) were risk factors associated with increased DTC susceptibility.

## Background

Thyroid cancer is classified as differentiated thyroid cancer and undifferentiated thyroid cancer according to histology. For further classification, differentiated thyroid cancer including papillary thyroid carcinoma and follictalar thyroid carcinoma, in which papillary thyroid carcinoma accounts for 75% in thyroid cancer [1–3]. In the past few years, these polymorphisms (rs965513, rs944289 and rs1867277) in the Forkhead factor E1(FOXE1) region and differentiated thyroid cancer (DTC) risk had been independently researched by subsequent studies [4–6]. The results and conclusions of these studies had been influenced by the use of different insufficient power, ethnic, phenotypic heterogeneity and small effect of the polymorphism on thyroid cancer risk and. In addition, with more Eastern Asian studies in recent years were reported, it is needed to reconcile these data.

FOXE1 gene is a specific thyroid transcription factor. It plays an important role in the growth and development of thyroid gland, the proliferation and differentiation of thyroid follicular cells. It is the key regulator of cellular function, including cell growth and differentiation, and plays an important role in the process of tumor development, invasion and metastasis. Therefore, this meta-analysis was conducted to make this discrepancy clear and to create a comprehensive picture of the association between common variants on FOXE1 and differentiated thyroid cancer.

Recently, significant progress has been made in the study of the association between DTC and susceptible genes through the genome-wide association strategy (GWAS). Single nucleotide polymorphisms (SNPs) related with DTC risk on chromosomes 5q24, 8q24, 9q22, and 14q13 had been identified by several recent studies [7–10]. The common genetic variation of FOXE1 has been identified as a new research hotspot in the susceptibility of differentiated thyroid cancer [11, 12]. The contribution of genetics to the risk of differentiated thyroid cancer was greater than to any other cancer. The identification and further assessment of the relevant genetic variations were important to know the potential mechanisms involved in differentiated thyroid carcinogenesis.

## Methods

### Literature search strategy

A comprehensive electronic literature search of the PubMed Database, EmBase Database, Cochrane Library and China National Knowledge Infrastructure (CNKI) from January 1981 to April 2016 was conducted. Two researchers carried out literature search independently. The search terms were as follows: [1] “rs965513” or “rs944289” or “rs1867277” or “FOXE1”; [2] “differentiated thyroid cancer” or “DTC”; [3] “association” or “associated”. These keywords were combined to seek for the researches using the Boolean operator “and” without languages restriction. In addition, the reference catalogues of all retrieved papers were checked for qualified articles which were not included as abovementioned. Then, we used Google Scholar (http://scholar. google.com/) to query the articles citing these studies and all of the references therein as identified using PubMed.

### Study Selection

The inclusion criteria were as follows: [1] the study must adopt a case-control design; [2] the study evaluated the relationship between rs965513, rs944289 or rs1867277 polymorphism and thyroid cancer; [3] the study provided the number of rs965513, rs944289 or rs1867277genotypes or provided sufficient data to calculate the number of rs965513, rs944289 or rs1867277 genotypes; [4] the study provided an Odds Ratio (OR) with 95% Confidential Interval (CI) as well as the *P* value or the study provided sufficient data to calculate the OR and 95% CI [5] at least 10 patients entered; [6] no lapping data was included.

Two authors assessed possibly related articles independently complying inclusion criteria and exclusion criteria. If there is disagreement between two researchers, a third author will help to solve it.

### Data extraction

The following information was extracted from every study: [1] the first author names; [2] the publication year; [3] the population or ethnicity; [4] the type of populations in cases and controls; [5] the numbers of cases and controls; [6] Genotyping method.

### Genetic model

The rs965513 polymorphism includes the two alleles G and A, of which A is the minor allele. A is assumed to be the high-risk allele and G the low-risk allele. The rs944289 involves the two alleles T and C, and T is the major allele which is reported to be the high-risk allele. In rs1867277, there existed two alleles G and A, in which A is the minor allele and assumed to be the high-risk allele. Additive, dominant, recessive genetic model were adopted for further meta-analysis. The additive model can be described as the A allele versus the G allele or the T allele versus the C allele.

### Statistical methods

The risk of differentiated thyroid cancer with the relative intensity of the associated intensity of FOXE1 polymorphism was evaluated by crude ORs value with the corresponding 95% CIs. Cochran's χ^2^ statistics [2] and I^2^ [7] were used on the assessment of the joint study of possible heterogeneity test. Generally speaking, when I^2^ value is 25%, it corresponded to no or little heterogeneity. The value 25 - 50% corresponds to the moderate heterogeneity, and the value 50% corresponds to the strong heterogeneity between studies. The random effect and the fixed effect are taken as the weighted average of the inverse variance of the logarithmic dominance ratio. The results of the random effect summary are reported in the text, because it takes into account the differences between the studies. Sources of heterogeneity were investigated by stratified meta-analyses based on ethnicity, sample size. Ethnic group was defined as East Asians, and Caucasians. Publication bias was assessed with the Begg’s and Egger’s t. Sensitivity analysis [10] is conducted by removing a person’s study from the total and analyzing the remainder. The analysis was conducted by STATA software version 10.0 (Stata Corporation, College Station, TX). All *P* values were two-side analysis, and 0.05 were considered statistically significant.

## Results

### Literature search

**Fig. 1:**
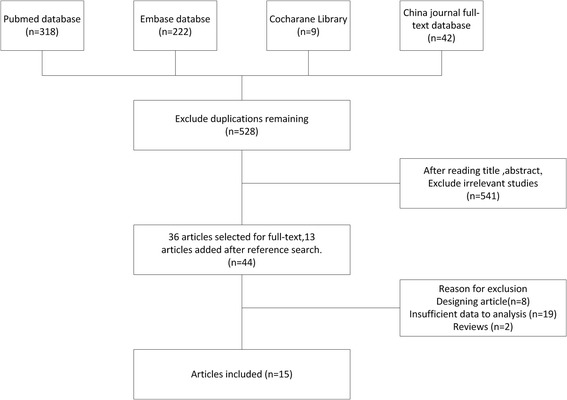
Flow gram of study selection

A total of 528 articles were initially searched out after the primary selection, and finally 15 researches [13–27] were included for the meta-analysis with 11895 DTC cases and 66385 controls (Fig. 1, Table 1). There are 13 data sets from 15 studies with 8,602 cases and 102,846 controls concerning rs965513, 11 data sets from 13 studies involving 2,017 cases and 13,281 controls concerning rs944289 and 7 data sets from 7 researches including 1,382 cases and 9,239 controls concerning rs1867277. Of the cases, 80% were Caucasian, and 20% were East Asian. Ten studies were of high quality, and five studies were of medium quality. No study of "poor quality" was found. The detailed features of the study included in this meta - analysis are shown in Table 1.

**Table 1:** Characteristics of the studies included in the meta-analysis

Study	Year	Polymorphism	Ethnicity	Cases	Controls	No. of cases/controls	Genotyping method	Quality	P for HWE
Francesca Damiola [21]	2013	rs965513, rs944289, rs1801516	France	Pathologically confirmed papillary thyroid cancer	General population	70/303	PCR, HRM, TaqMan	Median	0.6923
Angela Jones [22]	2011	rs965513, rs944289	Bratian	Histologically confirmed papillary thyroid cancer	General population	781/5193	TaqMan	High	0.3685
Celia Pereda [23]	2015	rs1801516, rs944289, rs965513	France	Pathologically confirmed papillary thyroid cancer	General population	203/212	TaqMan	Median	0.8552
Joshua Denny [24]	2011	rs965513	USA	Pathologically confirmed papillary thyroid cancer	Cancer-free individuals	1317/5053	TaqMan	High	0.4512
Julius Gudmundsson [25]	2009	rs965513, rs944289, rs1801516	Iceland	Histologically confirmed papillary thyroid cancer	Cancer-free individuals	962/38923	SNP arrays	High	0.3234
Stephane Maillard [26]	2015	rs944289, rs965513, rs1801516	France	Histologically confirmed papillary thyroid cancer	General population	168/262	TaqMan	Median	0.9704
Sandya Liyanarachchi [27]	2013	rs966425, rs944289	USA	Histologically confirmed papillary thyroid cancer	General population	2542/3137	SNP arrays	High	0.1188
Marissa Martinez [28]	2014	rs965513, rs944289	Germany	Histologically confirmed papillary thyroid cancer	General population	638/874	PCR, TaqMan	High	0.2126
Dorota Kula [29]	2016	rs965513, rs944289	Poland	Histologically confirmed papillary thyroid cancer	General population	2243/1160	PCR, TaqMan	High	0.3724
Michal Swierniak [30]	2015	rs965513, rs944289	Poland	Histologically confirmed papillary thyroid cancer	General population	1836/2313	SNP arrays	High	0.7532
Ana Florez [31]	2016	rs965513, rs944289	Colombia	Histologically confirmed papillary thyroid cancer	Cancer-free individuals	281/1146	PCR, KASP	High	0.7616
Liang Yu [32]	2017	rs966423, rs944289	China	Pathologically confirmed papillary thyroid cancer	Cancer-free individuals	349/1212	SNP arrays	High	0.1154
Liu Chen [33]	2013	rs965513, rs944289	China	Histologically confirmed papillary thyroid cancer	General population	152/183	PCR, TaqMan	Median	0.0001
Jiang Yongxin [34]	2014	rs966423, rs965513	China	Pathologically confirmed papillary thyroid cancer	General population	92/102	SNP arrays	Median	0.1334
Martyn Bullock [35]	2012	rs1801516		Pathologically confirmed papillary thyroid cancer	General population	261/6312	SNP arrays	High	0.2312

### Meta-Analysis

**Fig. 2:**
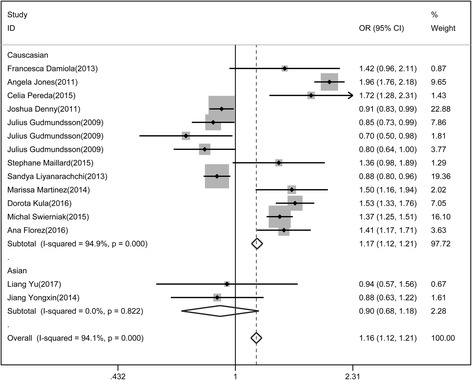
Association of rs965513 polymorphism with differentiated thyroid cancer

To analyze the association between rs965513 polymorphism and differentiated thyroid cancer, we performed the random effect model to calculate the total OR of the Caucasus population, and used the fixed effect model to analyze the heterogeneity in the East Asian population. The risk of thyroid cancer associated with the A allele was 1.16-fold that of the G allele (Fig. 2, OR = 1.16(1.12, 1.21). Moreover, we found that Caucasian populations had higher risk than East Asian populations (OR = 1.17 vs. 0.90). The association between rs965513 and thyroid cancer was also significant in the dominant model (AA + AG vs. GG, OR = 0.87(0.818, 0.926)), the recessive model (AA vs. AG + GG, OR = 1.269(1.199, 1.342)).

**Fig. 3:**
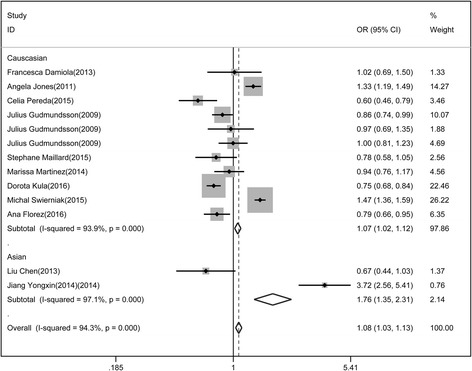
Association of rs944289 polymorphism with differentiated thyroid cancer

Similarly, a meta-analysis for the association of rs944289 polymorphism and differentiated thyroid cancer was conducted. Both Caucasian and East Asian populations were analyzed by random effect model. The risk of differentiated thyroid cancer of Allele model in Caucasian was higher than that in East Asian populations (Fig. 3, OR = 1.76 vs 1.07). The relationship of rs944289 polymorphism with differentiated thyroid cancer was statistically significant in dominant model (1.127(1.05, 1.21)) and recessive model (0.947(0.882, 1.017)).

**Fig. 4:**
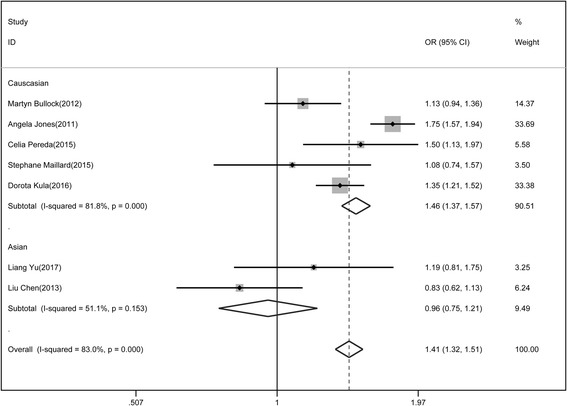
Association of rs1867277 polymorphism with differentiated thyroid cancer

Besides, the meta-analysis of association for rs1867277 and differentiated thyroid cancer was also conducted. In Caucasian populations, random effect model was adopted while in East Asian populations, fixed effect model was used. The risk of thyroid cancer associated with the A allele was 1.46-fold that of the G allele in Caucasian populations while that of the A allele was 0.96-fold that of the G allele in East Asian (Fig. 4). The association of rs1867277 with differentiated thyroid cancer was significant in dominant model (0.631(0.561, 0.709)) and recessive model (1.583(1.43, 1.753)).

### Heterogeneity test

The genetic heterogeneity of the rs965513 polymorphism was evaluated based on the additive, dominant and recessive models and the data from the selected studies (Table 2). The heterogeneity results of these 3 models were shown in Table 2. Significant heterogeneity was observed among these studies. In the additive model (A vs. G), the dominant model (AA + AG vs. GG) and the recessive model (AA vs. AG + GG), extreme heterogeneity was observed among the 15 selected studies (additive model: *P* < 0.0001 and I^2^ = 94.1%; dominant model: *P* < 0.0001 and I^2^ = 90.9%; recessive model: *P* < 0.0001 and I^2^ = 93.8%).

**Table 2:** Main results of overall and subgroups in the meta-analysis

Polymorphism	Subgroup	risk allele			dominant model			recessive model		
		OR(95%CI)	P	I^2^	OR(95%CI)	P	I^2^	OR(95%CI)	P	I^2^
rs965513	Total	1.162 (1.117,1.208)	0	94.10%	0.87 (0.818,0.926)	0	90.90%	1.269 (1.199,1.342)	0	93.80%
	Ethnicity									
	Caucasian	1.168 (1.122,1.215)	0.001	94.9	0.867 (0.815,0.922)	0	92.10%	1.284 (1.213,1.359)	0	94.40%
	East Asian	0.897 (0.680,1.193)	0.822	0	1.424 (0.716,2.830)	0.721	0.00%	0.679 (0.448,1.029)	0.085	66.40%
	Sample size									
	No.cases<500	1.320 (1.118,1.558)	0.029	66.60%	0.578 (0.403,0.83)	0.043	63.20%	1.92 (1.546,2.385)	0	89.00%
	No.cases≥500	1.153 (1.107,1.200)	0	95.60%	0.881 (0.827,0.938)	0	92.90%	1.231 (1.161,1.305)	0	94.50%
rs944289	Total	1.082 (1.035,1.131)	0	94.30%	1.127 (1.05,1.21)	0	88.60%	0.947 (0.882,1.017)	0	76.00%
	Ethnicity									
	Caucasian	1.067 (1.020,1.116)	0.001	93.9	1.151 (1.070,1.237)	0	84.30%	0.948 (0.882,1.02)	0	80.00%
	East Asian	1.764 (1.349,2.307)	0.001	97.1	0.548 (0.356,0.845)	0	96.90%	0.892 (0.584,1.363)	0.955	0.00%
	Sample size									
	No.cases<500	0.97 (0.837,1.125)	0	93.90%	1.129 (0.899,1.417)	0	91.30%	0.77 (0.604,0.981)	0.038	60.60%
	No.cases≥500	1.093 (1.044,1.145)	0	95.00%	1.127 (1.046,1.215)	0	88.10%	0.965 (0.896,1.04)	0	81.10%
rs1867277	Total	1.415 (1.324,1.512)	0	83.00%	0.631 (0.561,0.709)	0.012	63.50%	1.583 (1.43,1.753)	0	86.50%
	Ethnicity									
	Caucasian	1.463 (1.365,1.567)	0.001	81.8	0.605 (0.537,0.683)	0.114	46.40%	1.644 (1.479,1.827)	0	88.20%
	East Asian	0.957 (0.755,1.214)	0.153	51.1	1.354 (0.791,2.318)	0.464	0.00%	0.952 (0.647,1.4)	0.047	74.70%
	Sample size									
	No.cases<500	1.131 (0.945,1.353)	0.02	74.40%	0.923 (0.645,1.319)	0.142	48.90%	1.195 (0.918,1.555)	0.008	79.50%
	No.cases≥500	1.466 (1.365,1.574)	0	85.40%	0.601 (0.531,0.681)	0.06	59.50%	1.661 (1.487,1.856)	0	90.10%

In view of the significant heterogeneity and the search for its potential sources, we conducted a group of subgroups of race and sample size. When the race is stratified, the caucasians have a significant risk in all the comparisons. (risk allele: OR = 1.162(1.117, 1.208), *P*< 0.0001; dominant: OR = 0.867(0.815, 0.922), *P*<0.0001; recessive: OR = 1.284(1.213, 1.359), *P*<0.0001). However, no significant associations were observed for East Asians (risk allele: OR = 0.897(0.680, 1.193), *P*=0.822; dominant: OR = 1.424 (0.716, 2.83), *P*=0.721; recessive: OR = 0.678(0.448, 1.029), *P*=0.085). To take the population scape into consideration, the samples were divided into <500 and ≥500. No.cases<500 was small samples group and no.cases≥500 was large sample group. When the number of cases was considered, both no.cases<500 and no.cases≥500 were significantly associated. For less than 500 group, the results showed that risk allele: OR = 1.32(1.118, 1.558), *P*=0.029; dominant: OR = 0.578(0.403, 0.83), *P*=0.043; recessive: OR = 1.92(1.546, 2.385), *P*<0.0001). For more than 500 group, the results showed that risk allele: OR = 1.153(1.107, 1.20), *P*<0.0001; dominant: OR = 0.881(0.827,0.938), *P*<0.0001; recessive: OR = 1.231(1.161, 1.305), *P*<0.0001).

Similarly, in the heterogeneity test of the rs944289 polymorphism, three models were significant and OR were 1.082, 1.127 and 0.974 in allele risk, dominant model and recessive model respectively. Except recessive model in East Asian was insignificant, two other models in East Asian and three models in Caucasian were significant. In Caucasian, the OR values in allele risk, dominant and recessive models were 1.067, 1.151 and 0.948 respectively. In East Asian, the OR values in allele risk and dominant were 1.764 and 0.548 respectively. In the heterogeneity test of the rs1801516 polymorphism, three models were significant and OR were 1.415, 0.631 and 1.583 in allele risk, dominant model and recessive model respectively. Dominant models in Caucasian and East Asian were insignificant and risk allele in East Asian was insignificant. In risk allele model of Caucasian, OR value was 1.463. In Caucasian and East Asian populations, the OR values in recessive model were 1.644 and 0.952 respectively.

### Sensitivity and publication bias analysis

**Fig. 5:**
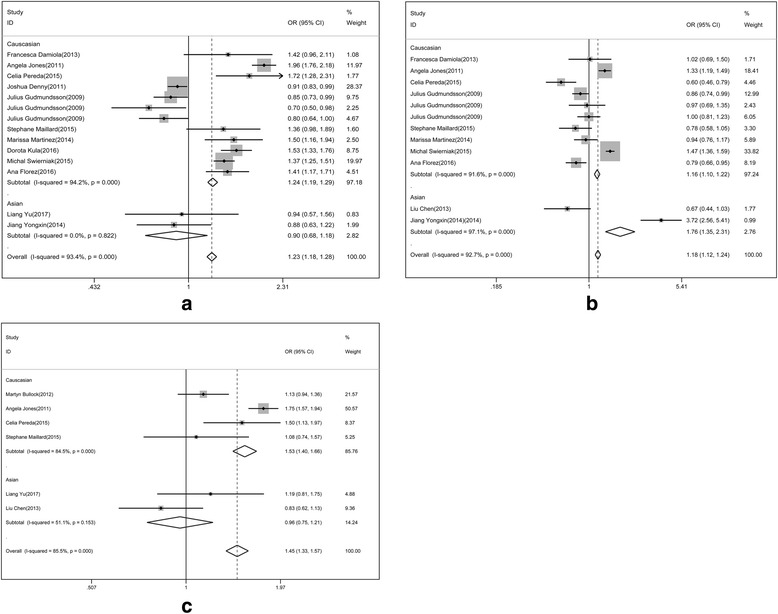
Sensitivity analysis of 3 SNPs: **a**. sensitivity analysis in rs965513, **b**. sensitivity analysis in rs944289, **c**. sensitivity analysis in rs1867277

**Fig. 6:**
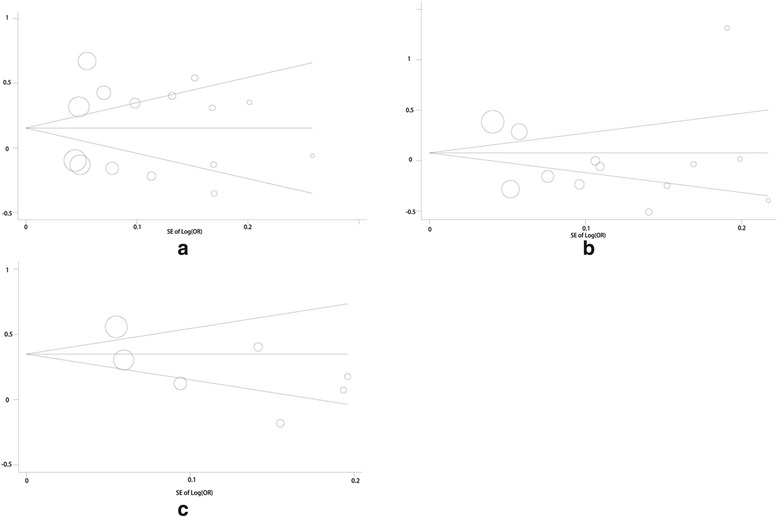
Funnel plots of 3 SNPs: **a**. funnel plot in rs965513, **b**. funnel plot in rs944289, **c**. funnel plot in rs1867277

Sensitivity analysis was carried out to assess the robustness of the meta - analysis results (Fig. 5). The pooled ORs from different populations were not influenced by removal of one study in three SNPs including rs965513, rs944289 and rs1867277, which supports the stability of this meta-analysis. Begg’s and Egger’s tests were conducted for publication bias evaluation. On the whole, the funnel plots of rs965513, rs944289 and rs1867277l were all symmetrical inverted funnels (Fig. 6). These results indicated that no significant publication bias existed in the meta-analysis. The above proved that the conclusions of our study were stable and credible.

## Discussion

It is reported that female, Asian, highly educated, history of goiter and family history of thyroid disease are risk factors for thyroid cancer. Zhu stated that differentiated thyroid cancer was a kind of disease which was influenced by several factors [28–30]. Compared with environment elements, genetic factors might be the main factors and lead to the difference of individual susceptibility. According to several reports, family risk rate of immediate family members in PTC could be 8%-12%, which is the highest one in all the cancers. The FOXE1 gene is a candidate gene associated with thyroid papillary carcinoma found by GWAS. FOXE1 (forhead box E1) was first isolated in the mouse cDNA library, also known as thyroid transcription factor 2 from the fork/spiro-wing structure of the transcription factor family. It is located on chromosome 9q22.23 and 14q33.3 containing only one exon, whose encoded protein consists of N-terminal region, highly conserved forkhead region, alpha helix poly-alanine channel and unique C-terminal residue. FOXE1 is one of the important tumor suppressor genes, and its relationship with malignant tumors has been explored for a long time. At present, many studies have found that FOXE1 gene played a role in skin squamous cell carcinoma, leukemia, pancreatic cancer, breast cancer, thyroid cancer and head squamous cell carcinoma and other tumors, which indicated that the research on FOXE1 was important. Liu reported that the distribution of rs944289 in FOXE1 in thyroid cancer patients and health population was different [2]. The risk of the T allele in Patients with TC was 4.84 –fold that of the C allele (OR=4.84, 95%CI = 1.597-14.668, *P*=0.005). However, Kang et al. evaluated the association of 12 SNPs in FOXE1 and PTC, and reported that rs965513 showed no association with PTC [10]. It is important to conduct a meta-analysis to assess the association between FOXE1 including rs965513, rs944289 and rs1867277 and differentiated thyroid cancer.

In this meta-analysis, the heterogeneity of rs965513 among the included studies was assessed, and there existed significant heterogeneity among the additive, dominant and recessive models. To further analyze the heterogeneity, stratified test was conducted and it showed that significant heterogeneity was observed among Caucasian populations but was not found in Asian populations. Meanwhile, both in no.case<500 and no.case≥500 groups, the heterogeneity was significant. These results may be resulted from that more studies corresponded to Caucasian populations, and the number of different case amount was similar. When studies were stratified for ethnicity, significant risks were found among Caucasians in all models, the A allele had a 1.1.62 fold risk. Compared with the dominant model, the recessive model showed the highest odds ratio in Caucasians populations (recessive: OR = 1.284(1.213, 1.359), *P*<0.0001). When the number of cases was considered, both no.cases<500 and no.cases≥500 were significantly associated. For less than 500 group, the A allele had a 1.32 fold risk. For more than 500 group, the results showed that the A allele had 1.153 fold risk. The sensitivity analysis suggested that the results of this meta-analysis were stable. For short, the Caucasian populations had higher risk than East Asian populations. These were consistent with several previous researches [31, 32].

In addition, rs944289 among the selected studies had significant heterogeneity. The heterogeneity in the additive, dominant and recessive models were significant. The stratified results showed that three models in Caucasian and East Asian were significant. The risk of the T allele was 1.067 fold (1.020, 1.116) in Caucasian populations and that was 1.764 fold (1.349, 2.307) in East Asian populations. In No.cases <500 group, the T allele had 0.97 fold risk (0.837, 1.1.25) and in No. cases≥500 group, the T allele had 1.093 fold risk (1.044, 1.145). In conclusion, in rs944289, East Asian had more risk than Caucasian populations.

At last, the research about heterogeneity in rs1867277 was carried out. Through subgroup analysis, we divide race into a potential source of heterogeneity. In the race stratified analysis, it is observed that association between rs1867277 polymorphism and risk for DTC in Caucasians (OR = 1.463, 95%CI=1.365, 1.567) was stronger than that in East Asian populations (OR = 0.957, 95%CI=0.755, 1.214). In the tratified analysis by number of cases, that association between rs1867277 polymorphism and risk for DTC in No.cases<500 (OR = 1.131, 95%CI=0.945,1.353) was stronger than that in No.cases≥500 (OR = 1.466, 95%CI=1.365, 1.574). Therefore, the Caucasian populations had higher risk than East Asian populations concerning rs1867277. These conclusions were similar with some previous studies [33–35].

In inferring the results, some limitations of this meta-analysis should be added. At first, in this study, the majority of subjects are of European descent, so statistical power for analyses in other ethnicities is limited. This is due to the small sample size in East Asia studies. Therefore, The main conclusion of the manuscript is based on the analysis between the Caucasus. In the further studies, more East Asians or Africans are needed to clarify the consistency found by different ethnic groups. Second, meta-analysis is a kind of retrospective study, which might lead to the recall and selection bias.

Despite these limitations, this meta-analysis suggested that the three common variations on FOXE1 (rs965513, rs944289, rs1867277) had significant relationship with the increase in the risk of DTC, particularly in Caucasian population. Since the study of other ethnic groups is still limited, further studies will include a wider range of subjects to investigate the role of these variants in other populations. Besides, the possible gene-gene and gene-environmental interactions in this association would be explored in the further researches.

## Conclusion

This meta-analysis suggested that common variations of FOXE1 (rs965513, rs944289 and rs1867277) were risk factors associated with increased DTC susceptibility.

## Abbreviations

DTC: Differentiated thyroid carcinoma
GWAS: Genome-wide association strategy
SNPs: Single nucleotide polymorphisms
